# Palliative and End-of-Life Care: More Work is Required

**DOI:** 10.3390/ijerph17207429

**Published:** 2020-10-13

**Authors:** Doris Y. P. Leung, Helen Y. L. Chan

**Affiliations:** 1 School of Nursing, The Hong Kong Polytechnic University, Hong Kong, China; 2 The Nethersole School of Nursing, Faculty of Medicine, The Chinese University of Hong Kong, Hong Kong, China; helencyl@cuhk.edu.hk

**Keywords:** palliative care, end-of-life care, policy

## Abstract

There is currently growing recognition of the complex care needs of patients with life-limiting conditions and their family members, prompting the need to revisit the goals of medicine. This Special Issue reflects a broad research agenda in the field of palliative and end-of-life care. A total of 16 papers of empirical studies and systematic review are included spanning five domains, namely, patient, caregiver, healthcare provider, policy, and methodology. The results generally suggest the merits of palliative care and reveal room for further improvement in palliative care education, manpower, infrastructure, and legal and policy frameworks.

## 1. Introduction

The landscape of palliative and end-of-life care has changed substantially in the last decade due to changes in demographics, medical technologies, and disease patterns, resulting in a huge number of people who live with chronic progressive conditions requiring palliative care [1]. Decisions over medical treatments are becoming increasingly complicated, as survival rate is no longer seen as the only care outcome and the burdens and benefits resulting from treatments are subject to interpretation, giving rise to growing concerns over what is meant by a “good death” and how to define good care near the end of one’s life. The increasing number of people requiring palliative care is also leading to an unprecedented dependence on carer support for high-quality care [1]. Palliative care emphasizes symptom control and meeting the complex care needs of patients with life-limiting conditions and their families to optimize quality-of-life [2]. There is a growing realization that the provision of palliative care is a vital component of healthcare regarding addressing various distressing symptoms and social and psychospiritual concerns from the time of diagnosis until the last phase of life [3].

## 2. Included Works

This Special Issue intends to inform policy and practice regarding the improvement of palliative and end-of-life care. The articles included reflect multifaceted interests broadly classified into five domains, namely, patient, caregiver, healthcare provider, policy, and methodology, although the studies overlap across some domains (Table 1). The methods of the accepted publications varied, comprising quantitative cross-sectional and longitudinal studies, qualitative studies, psychometric instrument validations, and systematic reviews. The majority of the primary studies (*n* = 14) were conducted in Asia (*n* = 8) and Europe (*n* = 4), with one in South America and one in North America.

Five articles focused on the patient. A wide spectrum of patient populations was included, ranging from cancer patients to noncancer patients and older adults with moderate–severe physical impairment and cognitive frailty. Engel and colleagues [4] reported the establishment of a palliative care consultation service in an acute hospital in Germany. This palliative care consultation team supports physicians and nurses by providing medication recommendations for their patients. Using data from the medical records of inpatients and outpatients of the participating departments in the hospital, the study showed a significantly shorter mean time to referral in departments using the palliative care consultation service, although only 50% of the recommendations by the palliative care consultation team were accepted. Another innovation of this study was the use of a big data analytics heatmap to describe the symptom burden of patients, which could potentially be used by physicians and nurses to predict and prepare for the distinct and most frequent symptoms of palliative care patients in their departments.

Two studies identified factors of survivorship of palliative care patients using data from routine records. Cheng and colleagues [5] used data from the medical records of 305 patients with advanced cancer attending the Emergency Departments of three hospitals in Taiwan, showing the strong predictive power of a simple index, named the Shock index (pulse rate/systolic blood pressure), in predicting 60-day survival rate. Kawakami and Hamano [6] analyzed data from 106 residents with moderate-to-severe impairment of activities of daily living (ADL) and cognitive frailty in a nursing home in Japan, showing that change in body mass index, energy intake, and fluid intake were prognostic factors for death. Both studies highlighted the importance of the use of the simple evaluation tools based on routine measurements in identifying patients near end-of-life so that physicians in these settings can initiate advanced care planning discussions with identified patients and their families in a timely manner.

The other two studies in the patient category focused on healthcare utilization. Kao et al. [7] analyzed population-based data from national insurance records to discover the patterns of healthcare utilization in chronic obstructive pulmonary disease (COPD) patients with and without cancer who received palliative care in Taiwan. Significantly higher rates of intensive care unit (ICU) admissions, longer ICU stays, more bronchoscopic interventions, and more inpatient physical restraints were reported in COPD patients without cancer, whereas significantly more blood transfusions and CT scans were reported in COPD patients with cancer. Cerni and colleagues [8] carried out a systematic review to synthesize evidence regarding the disparity in end-of-life cancer care resource use for adult patients with cancer according to rurality. Based on 24 included studies, rurality was found to be strongly associated with higher rates of Emergency Department visits and hospitalization and lower rates of hospice care. The findings from both studies stressed that disparities in end-of-life resources remain according to patient group and rurality, and further efforts to reduce disparities for efficient and equitable palliative care are needed.

Two studies focused on the carer. Leung and colleagues [9] carried out the validation of the Support Person’s Unmet Needs Survey Short Form to measure the unmet needs of family caregivers taking care of cancer patients in the Hong Kong Chinese population. Using confirmatory factor analysis (CFA), the five-factor structure of the original scale, consisting of information, future concerns, work and finances, healthcare access and continuity, and personal and emotional needs, was replicated. Acceptable reliable and validity of the scale were evident, with internal consistency by ordinal alpha, stability by intraclass correlation coefficient, and concurrent validity by correlations, with related measures including caregiver burden and sleep disturbance. The scale could be used to identify Chinese carers of patients with cancer with unmet supportive needs, thereby allowing timely and appropriate interventions to be made to address specific unmet needs. Leung et al. [10] performed a path analysis on data from 225 patient-caregiver dyads to test the role of family and friend support and caregiving self-efficacy on caregiver burden and patient quality-of-life among Chinese patients with palliative care needs and their caregivers, with results showing that family support moderated the relationships of caregiving self-efficacy with caregiver burden and patient quality-of-life, whereas friend support showed a directly positive effect, instead of the expectation of a negative effect, on caregiver burden. Therefore, providing support from appropriate sources may be important in palliative care settings.

Four studies were classified into the category of healthcare workers in palliative and end-of-life care. Lehto et al. [11] interviewed 19 hospice workers in various professions in the US to solicit their views regarding their working environments in hospice organizations. Three main themes, namely, rewarding aspects, perceived challenges, and strategies to combat burnout and manage stress at the personal and organization levels, were identified. The authors stressed the usefulness of the information of the lived experience of hospice workers in informing the development of strategies to enhance employee well-being at the organizational level. Fernández-Martínez and colleagues [12] tested the reliability and validity of the Attitude Toward Euthanasia Scale for health workers within a Spanish population. Data from 201 health workers were subjected to exploratory and confirmatory factor analyses and reliability analyses. The Spanish version of the instrument was shown to have a new, more clear-cut, four-factor structure and demonstrated good internal consistency compared to the original factor structure with cross-factor items. The authors concluded that although the Spanish version of the instrument showed appropriate psychometric properties, more work is needed to replicate these findings.

The other two studies in this category related to palliative care undergraduate education. Pieters et al. [13] conducted a Delphi study aimed to validate a national palliative care competency framework for undergraduate medical curricula in the Netherlands. Using five groups of stakeholders and after two rounds of iteration, a high level of consensus regarding the ratings of six key competency domains was reached, with communication and personal development and well-being scoring the highest and advance care planning the lowest. This competency framework for palliative care could form a basis to integrate palliative care into the existing undergraduate medical curriculum. Noguera et al. [14] developed and validated an instrument, Student’s Inventory of Professionalism, assessing attitudes toward professional development based on palliative care undergraduate education. Using two cohorts of medical students in Spain, the final version of the instrument consisted of 33 items measuring seven domains and demonstrated good psychometric properties, including good internal consistency, acceptable factor structure and construct validity, and good matching with the elements of professionalism. The scale could be useful to assess student professional development in palliative care teaching.

Four studies focused on policy regarding palliative and end-of-life care. Dávalos-Batallas et al. [15] analyzed data from individual, semistructured interviews of 28 physicians to inform policy and practice regarding palliative care development in Ecuador. Five core themes were identified, including training, health policy, professionals’ activities, health services, and development of palliative care. The authors stressed the importance of palliative care training in countries in South America, particularly Ecuador, as no official training on palliative care for healthcare professionals and strategies to intensify such training are required. Wong et al. [16] solicited views from 72 health professionals from various disciplines to explore enabling and inhibiting factors in the implementation of palliative and end-of-life care for the elderly in Hong Kong. Using the Grounded theory to guide the analysis, a thematic framework consisting of four main themes, i.e., political context, organizational setting, support to patients, caregivers, and family members, and health workers and public were identified. The authors recommended three aspects for further development in the service implementation, including standardization of terminology across the setting, the use of a multidisciplinary approach and information sharing across the setting, and providing training to equip care providers with the necessary readiness and competency. Chan et al. [17] interviewed 131 participants including patients with life-limiting conditions, family members, health care providers, administrators, lawyers, and policy makers in Hong Kong. Analyzing the multiple stakeholders’ perspectives with the political-economic-social-technological-environmental-legal (PESTEL) framework for situation analysis, the authors identified a number of barriers and challenges, including policy, economic, sociocultural, environmental, and legal challenges, that hinder palliative and end-of-life care development. The authors concluded that an overarching theme is the need to formulate a government-led policy framework to achieve a paradigm shift relating to society as a whole to improve palliative and end-of-life care. Chung et al. [18] also conducted a multimethod qualitative study from patients’ and families’ perspectives to explore service gaps in end-of-life care in the older population in Hong Kong. Using a deductive thematic analysis, multilevel gaps and issues of end-of-life care at the policy, legal, community, institutional, intrapersonal, and interpersonal levels were identified, concluding that a lack of overarching end-of-life care policy framework exists. The authors emphasized the importance of cross-sectoral service integration, because end-of-life care-related policy should be an integral part of a long-term care policy.

One study was classified in the methodology category. Fàbregues et al. [19] identified and reviewed the methodological quality of 159 out of 3225 studies (4.9%) published between 2014 and 2019 in eight palliative care journals, reporting a mixed study design in palliative care research. Using the Good Reporting of a Mixed Methods Study criteria for assessment, 66.7% of the studies reported evidence of integration between the two methods, but only 3.8% reported the limitations of using one method associated with the presence of the other method and 26.4% described the insights gained from integration methods. The authors stressed that although there is a consensus regarding the usefulness and importance of a mixed-method approach in answering the complex questions in palliative care and end-of-life research, empirical articles utilizing this approach in this area are still scarce and the quality of reporting is poor, threatening the generation of robust, relevant, and transferable evidence for policy and practice in palliative care.

## 3. Conclusions

The studies presented in this Special Issue cover a wide range of issues related to different aspects in the field of palliative and end-of-life care. They document not only the progress being made in different pathways to improve the practices of care and the development and implementation of the related policies in different countries, but also the high complexity of the challenges faced to answer complex questions regarding palliative and end-of-life research. A global shift in the healthcare paradigm from a biomedical-oriented to a holistic approach in multiple and overlapping dimensions is evident, but the progress is still in its infancy stage. Frequent updates from multiple stakeholders could lead to a concerted effort for smoother transition to high-quality palliative and end-of-life care in the future.

## Figures and Tables

**Table 1:** Description of the articles included in the Special Issue by research domain.

Authorship	Study Focus	Country	Methodology	Main Analyses	Main Findings
*Disease-specific patient groups*					
Engel et al. [4]	Patients with advanced life-limiting diseases in acute hospital	Germany	Quantitative, cross-sectional using hospital records	Heatmap and bivariate analyses	Realization of recommendations of drug conversions for symptom management by Palliative Care Consultation Service ranged from 32.1% to 59.4%.
Cheng et al. [5]	Patients with advanced cancer in the Emergency Department	Taiwan	Quantitative, longitudinal using hospital records	Multivariate logistic regression	A simple index (pulse rate/systolic blood pressure) was found to be useful in predicting 60-day survival rate.
Kawakami and Hamano [6]	Older adults with moderate-to-severe impairment of ADL and cognitive frailty	Japan	Quantitative, longitudinal using hospital records	Receiver operating characteristics and multivariate logistic regression	Changes in BMI, energy intake, and fluid intake over 60 months were found to be prognostic factors for death.
Kao et al. [7]	Palliative care patients with chronic obstructive pulmonary disease	Taiwan	Quantitative, cross-sectional using insurance data	Multivariate logistic regression	COPD patients without cancer appeared to receive more invasive healthcare interventions than COPD patients with cancer.
Cerni et al. [8]	Cancer patients receiving end-of-life palliative care	Canada, USA, Taiwan, Australia, Germany	Systematic review of quantitative studies	Narrative synthesis	Based on findings from 24 studies, rurality was strongly associated with higher rates of Emergency Department visits and hospitalizations and lower rates of hospice care.
*Family caregivers of patients with life-limiting condition*					
Leung et al. [9]	Caregivers of cancer patients	Hong Kong	Psychometric instrument validation	Confirmatory factor analysis, reliability analyses and Pearson correlations	The original five-factor model was replicated and good reliability and construct validity were obtained.
Leung et al. [10]	Caregivers of patients with palliative care needs	Hong Kong	Quantitative, cross-sectional	Path analysis	Caregiving self-efficacy showed a directly negative effect on caregiver burden and a directly positive effect on patient’s quality of life. Family support showed a positive direct effect on caregiving self-efficacy while friend support has a positive direct effect on caregiver burden.
*Healthcare providers in palliative care services*					
Lehto et al. [11]	Hospice care workers	USA	Qualitative, focus group, and semi-structured interviews	Constant comparative analysis	Benefits of hospice caregiving included intrinsic satisfaction from the work, receiving positive patient and family feedback, and teamwork. Challenges included workload, technology issues, administrative demands, travel-related problems, communication and interruptions, difficulties with taking time off from work and maintaining work-life integration, and coping with witnessing grief/loss.
Fernández-Martínez et al. [12]	Healthcare workers	Spain	Psychometric instrument validation	Exploratory and confirmatory factor analyses and Cronbach’s alpha	A four-factor structure consisting of severe pain, no recovery, patient request, and doctor’s authority was obtained.
Pieters et al. [13]	Healthcare professionals and educators	Netherlands	Qualitative, Delphi study	Bivariate analysis	A competency framework for palliative care education was developed, with communication, personal development, and well-being scoring the highest.
Noguera et al. [14]	Medical students	Spain	Psychometric instrument validation (second part of a sequential mixed method)	Exploratory and confirmatory factor analyses and reliability analyses	A tool measuring student attitudes toward professional development in palliative care was developed consisting of seven dimensions of holistic care, care and understanding, personal growth, teamwork, decision-making, patient assessment, and being a healthcare professional.
*Policy related to palliative care services*					
Dávalos-Batallas et al. [15]	Policy	Ecuador	Qualitative, individual semistructured interviews	Thematic analysis	Five core themes including training, health policy, professionals’ activities, health services, and development of palliative care.
Wong et al. [16]	Policy	Hong Kong	Qualitative, focus group, and individual interviews	Grounded theory	Quality palliative care provision was found to be influenced by the interaction and integration of four themes, including political context, organization setting, support to patients, caregivers, and family members, and healthcare workers and the public.
Chan et al. [17]	Policy	Hong Kong	Qualitative, focus group, and individual interviews	Situation analysis	Palliative and end-of-life care service development involved a paradigm shift relating to society as a whole. The overarching theme was to formulate a government-led policy framework.
Chung et al. [18]	Policy	Hong Kong	Qualitative, focus group, individual interviews, and longitudinal case studies	Deductive thematic analysis based on a socioecological model	Multi-level service gaps at the policy, legal, community, institutional, intrapersonal, and interpersonal level were identified.
*Methodology*					
Fàbregues et al. [19]	Methodology	NA	Systematic review of empirical mixed-method studies	Content analysis	Included studies (n = 159) usually did not describe the type of mixed-method design used and provided little detail on the integration of quantitative and qualitative methods.

ADL: activities of daily living; BMI: body mass index.
